# Fatty acid synthesis: A critical factor determining mycelial growth rate in *Pleurotus tuoliensis*

**DOI:** 10.1016/j.fochms.2025.100285

**Published:** 2025-08-13

**Authors:** Yu Tian, Suyue Zheng, Jinwei Zhang, Qiang Chen, Ruiying Zhang

**Affiliations:** a State Key Laboratory of Efficient Utilization of Arable Land in China the Institute of Agricultural Resources and Regional Planning, Chinese Academy of Agricultural Sciences, Beijing 100081, China; b Key Laboratory of Microbial Resources Collection and Preservation, Ministry of Agriculture and Rural Affairs, Beijing 100081, China; c School of Landscape and Ecological Engineering, Hebei University of Engineering, Handan 056038, China

**Keywords:** Edible mushroom, *Pleurotus tuoliensis*, Acetyl-CoA carboxylase, Fatty acid, Lipid

## Abstract

*Pleurotus tuoliensis* is a commercially important edible mushroom renowned for its delicious taste and health benefits. Compared with other cultivated species in the genus *Pleurotus*, the vegetative mycelium of *P. tuoliensis* grows relatively slowly. In this study, the *PtACC* gene of *P. tuoliensis*, which encodes acetyl-CoA carboxylase, was cloned. The expression level of *PtACC* was regulated by RNA interference (RNAi) and overexpression methods. The results showed that RNAi reduced the expression level of *PtACC*, which directly decreased the total lipids content and the mycelial growth rate. Overexpression increased the expression level of *PtACC*, directly increasing the total lipid content and the mycelial growth rate. However, if the expression level of *PtACC* increased by more than double, the significant increase in the total lipid content would instead inhibit mycelial growth. Adding bicarbonate and ammonium acetate, which are reaction substrates of PtACC, to PDA medium could also increase the total lipid content and the mycelial growth rate. The optimal pH for the mycelial growth of *P. tuoliensis* is between 8.0 and 9.0, and the highest total lipid content of the mycelium is achieved at pH 8.0. In addition, adding fatty acids such as stearic acid (C18:0), oleic acid (C18:1n9c), linoleic acid (C18:2n6c), and docosanoic acid (C22:0) to PDA medium separately could also increase the mycelial growth rate. These findings indicate that *PtACC* is a key gene controlling lipid synthesis and mycelial growth in *P. tuoliensis*.

## Highlights

• Increasing ACC enzyme substrate content in medium promotes lipid synthesis and mycelial growth.
• ACC gene expression determines mycelial growth rate by regulating lipid synthesis.
• Fatty acid synthesis is an important factor affecting P. tuoliensis mycelial growth rate.

## 1 Introduction

*Pleurotus tuoliensis*, commonly known as Bailinggu, is a commercially important edible mushroom cultivated in many countries, prized for its white fruiting body, crisp texture, and delicate taste. Wild populations of *P. tuoliensis* are predominantly found in Xinjiang, China. The species was first documented in 1987 when Mou et al. collected specimens from Tuoli County, Xinjiang, initially describing it as a new variety of *Pleurotus eryngii*, *P. eryngii* var. *tuoliensis* (Mou et al., 1987). *P. tuoliensis* was domesticated successfully in the early 1980s, and commercial cultivation scaled up in the late 1990s in China, driven by its dual significance in nutrition and traditional medicine (Jia et al., 2024; Jia & Qin, 2006). However, it was erroneously referred to as *Pleurotus nebrodensis* in many literatures for decades (Dai & Yang, 2018). This confusion ended in 2016 when it is confirmed as a distinct species, *P. tuoliensis*, according to phylogenetic analysis using molecular markers (ITS/LSU rDNA) (Zhao et al., 2016). According to the China Edible Fungi Association, annual production has grown steadily, with nationwide output exceeding 1.2 million tons in 2023.

Compared with other cultivated species in the genus *Pleurotus*, *P. tuoliensis* exhibits strikingly large fruiting bodies, with individual weights typically exceeding 400 g. In contrast, its vegetative mycelium is exceptionally slender and displays slow growth rate. To increase mycelial growth rate and fruiting body yield in commercial cultivation for edible mushrooms, the previous researches has predominantly focused on optimizing the carbon‑nitrogen (C/N) ratio of culture substrates and screening for optimal carbon/nitrogen sources (Hu et al., 2022; Ma et al., 2021; Osunde et al., 2019; Wang et al., 2021). For carbon sources, investigations centered on identifying effective carbohydrate types and determining optimal concentrations, with studies showing that the most suitable carbon source is glucose, with a concentration ranging from 20 to 30 g/L for *P. tuoliensis* (Qiu & Wang, 2008). Up to now, little attention has been paid to the effects of lipid substances on the mycelial growth, fruiting body yield and quality of edible mushrooms.

Lipid substances play multiple important roles in the growth and development of fungi. Lipids such as glycerophospholipids, sphingomyelins, and glycolipids are the fundamental building blocks of fungal cell membranes, as well as the membranes of organelles such as the nucleus, endoplasmic reticulum, Golgi apparatus, and mitochondria etc. (Bergia & Rippa, 2025; Li et al., 2025; Rella et al., 2016). Some lipids such as farnesol and eicosanoids serve as crucial cellular signaling molecules, involved in physiological processes such as growth, development, morphogenesis, stress responses, and pathogenicity (Reverberi, 2019; Rhome & Del Poeta, 2009). Additionally, triacylglycerols (TAGs) represent the primary energy storage form in fungal cells. In oleaginous species such as *Mucor circinelloides* and *Yarrowia lipolytica*, lipid accumulation can reach upwards of 20 % of cellular dry weight, enabling cells to withstand nutrient-deprived conditions (Duman-Özdamar et al., 2025; Hassane et al., 2024). Given their indispensable roles, key enzymes within the lipid biosynthetic pathway have emerged as attractive targets for antifungal drug development. Promising compounds such as firsocosta and cerulenin, which inhibit lipid synthesis, have garnered significant attention (Gutierrez-Perez & Cramer, 2025; Vishwakarma et al., 2024). Collectively, these findings underscore the critical importance of lipids in fungal biology, highlighting their essentiality for fungal growth, development, and survival.

Acetyl-CoA carboxylase (ACC) catalyzes the rate-limiting step in de novo fatty acid synthesis (FAS) across all organisms. This enzyme utilizes bicarbonate, ATP, acetyl-CoA, and the biotin cofactor to generate malonyl-CoA, which serves as the building block for fatty acid synthesis (Salie & Thelen, 2016; Zhou et al., 2024). There are two major types of ACC: homomeric and heteromeric (Niazi & Moorhead, 2025). The heteromeric ACC is composed of four distinct subunits: biotin carboxylase (BC), biotin carboxyl carrier protein (BCCP), and α- and β-carboxyltransferases (CTs). Heteromeric ACC is predominantly found in bacteria and plastids of plants (Salie & Thelen, 2016). In contrast, homomeric ACCase consists of two identical polypeptide chains, with each chain containing three functional domains: the biotin carboxylase domain (BC), the biotin carboxyl carrier domain (BCCP), and the carboxyl transferase domain (CT) (Pereira et al., 2022). Homomeric ACCase exists as a homodimer to exhibit biological activity and is the predominant form in the cytosol of plant, animals and fungi. In *Saccharomyces cerevisiae*, two distinct acetyl-CoA carboxylase (ACC) isozymes have been identified. ACC1, a cytosolic enzyme, is essential and necessary for the de-novo synthesis of lipids. Conversely, HFA1, localized within the mitochondria, is essential for cellular respiration, probably due to a role in the synthesis of lipoic acid (Hasslacher et al., 1993; Hoja et al., 2004). However, for most fungi, there is only a single ACC, which is located in the cytoplasm.

Genetic engineering has emerged as a pivotal strategy for enhancing the production of fatty acids and their derivatives in oleaginous fungi. In *S. cerevisiae*, overexpression of endogenous gene acetyl-CoA carboxylase (*Acc1*) successfully increased the synthesis of fatty acid-derived biofuels and chemicals (Runguphan & Keasling, 2014), and heterologous expression of *Acc* from *Lipomyces starkeyi* significantly enhanced lipid accumulation (Wang et al., 2016). Similarly, in *Y. lipolytica*, *Acc* overexpression effectively boosted fatty acid and biodiesel yields (Duman-Özdamar et al., 2025; Xu et al., 2016), highlighting the central role of *Acc* in fatty acid biosynthesis. Complementary strategies focus on augmenting substrate availability. Overexpression of ATP-citrate lyase (*Acl*), carnitine acetyltransferase (*Cat*2), pantothenate kinase (*Pan*K), and genes of the pyruvate dehydrogenase bypass including pyruvate decarboxylase (*Pdc*), acetaldehyde dehydrogenase (*Acdh*), and acetyl-CoA synthetase (*Acs*), increases cytoplasmic acetyl-CoA levels, thereby promoting lipid synthesis (Liu et al., 2017; Wang et al., 2024; Xu et al., 2016). Additionally, enhancing bicarbonate ion concentration via carbonic anhydrase (*Ca*) overexpression has been shown to stimulate fatty acid production (Chadwick & Lin, 2024; You et al., 2021). Collectively, these previous studies demonstrate that both enhancing ACC activity and increasing substrate concentrations represent effective genetic modification approaches for optimizing fatty acid production in oleaginous fungi.

This study aimed to elucidate the impact of fatty acid synthesis levels on the growth rate of *P. tuoliensis* vegetative mycelia. By changing the expression of acetyl-CoA carboxylase (*Acc*) through overexpression and RNA interference (RNAi), we found that *Acc* levels directly influenced the total lipid content and mycelia growth rate. Additionally, increasing ACC substrate concentrations by adding ammonium acetate and sodium bicarbonate to PDA medium also increase lipid synthesis and mycelial growth. The study confirms that the lipid synthesis rate is a key limiting factor for mycelial growth, providing a theoretical basis for cultivation and industrial production. This study establishes a theoretical foundation for genetic breeding and delivers technical support for cultivation practices.

## 2 Materials and methods

### 2.1 Strain and medium

*P. tuoliensis* strain CCMSSC00489, provided by the China Center for Mushroom Spawn Standards and Control (CCMSSC). The strain was cultured on potato dextrose agar (PDA) medium and preserved using liquid nitrogen cryopreservation technology. To assess the effects of ammonium acetate on mycelial growth and lipid biosynthesis, the PDA media were supplemented with ammonium acetate at five distinct concentrations (5 mM, 10 mM, 20 mM, 40 mM, and 80 mM). To explore the effects of pH on mycelial growth and lipid biosynthesis, the pH of the PDA medium was adjusted using 1 mol/L HCl and 1 mol/L NaOH. To evaluate the impact of bicarbonate on mycelial growth and lipid biosynthesis, sodium bicarbonate at five different concentrations (5 mM, 10 mM, 20 mM, 40 mM, and 80 mM) was added to the PDA medium, with the pH of the medium maintained at 7.0. To investigate the effects of fatty acids on mycelial growth, five concentrations (0.05 mM, 0.1 mM, 0.5 mM, 1 mM, and 2 mM) of the following fatty acids were added to PDA medium: stearic acid (C18:0), oleic acid (C18:1n9c), linoleic acid (C18:2n6c), and behenic acid (C22:0).

### 2.2 DNA/RNA extraction and cDNA synthesis

The PDA medium plates with a diameter of 90 mm were inoculated using mycelial plugs with a diameter of 5 mm, and incubated in the dark at 25 °C for 7 days. Subsequently, the mycelium was harvested and ground into powder using liquid nitrogen. Genomic DNA (gDNA) was extracted with the Plant Genomic DNA Extraction Kit DP305–03 (Tiangen, Beijing, China). RNA isolation was performed using the Plant RNA Mini Extraction Kit R6827–01 (Omega, Norcross, GA, USA). Then, cDNA was synthesized from RNA templates using the Reverse Transcription Kit R312–02 (Vazyme, Nanjing, China). The above three experiments were carried out according to the instructions of the kits. All DNA and cDNA materials were stored at −20 °C, RNA materials were stored at −80 °C.

### 2.3 Gene cloning and plasmids construction

The genomic sequence of *PtACC* gene was amplified by PCR with the primer pair PtACC-F/R using *P. tuoliensis* gDNA as the template. The CDS fragment of the *PtACC* was amplified with the primer pair OE-PtACC-F/R from *P. tuoliensis* cDNA. The primers used in this study are listed in Table 1.

**Table 1:** The primers used in this study.

Primer	Sequence(5′ → 3′)
PtACC-F	ATGTCTGCATACGATCATAGCA
PtACC-R	CTACGACGTCCCGTATGATGGG
M13–20	TGTAAAACGACGGCCAGT
M13–26	CAGGAAACAGCTATGACC
OE-PtACC-F	GGTCAAAGTTACTAGTATGTCTGCATACGATCATAGCA
OE-PtACC-R	CAATTCTAGAGGGCCCCTACGACGTCCCGTATGATGGG
RNAi-sense-PtACC-F	CCATCTCCTCAGATCTACCTGGAAGCGCAATGAG
RNAi-sense-PtACC-R	TAAGCTCTAAACTAGTACCTTTGGAAGCGACGGG
RNAi-anti-PtACC-F	TTCCAAAGGTACTAGTACCTGGAATCTCTCCTGCGAC
RNAi-anti-PtACC-R	CAATTCTAGAGGGCCCACCTGGAAGCGCAATGAG
Hyg-F	TCGGTTTCCACTATCGGCGAGTACTTCTACACA
Hyg-R	TCTCGTGCTTTCAGCTTCGATGTAGGAGGG
qPtACC-F	TGGAGATCATGTCAACGCTG
qPtACC-R	GTCAATATACCAAGAATGTCGCC
qGAPDH-F	TGGCCCGTCGCATAAGGA
qGAPDH-R	ACACGGAAGGACAAACCA

The PCR products were analyzed by 1 % agarose gel electrophoresis and then purified using FastPure Gel DNA Extraction Mini Kit DC301–01 (Vazyme, Nanjing, China). The purified DNA fragments were ligated into the pEASY-Blunt Zero Cloning Vector CB501–02 (Vazyme, Nanjing, China) and transformed into *E. coli* DH5α (Vazyme, Nanjing, China). The cloned DNA fragments were sequenced with primers M13–20 and M13–26 Sangon Biotech (Shanghai) Co., Ltd.

In order to construct the RNA interference (RNAi) plasmid for the *PtACC* gene, a 350-bp sense fragment adjacent to the 5′-end of the *PtACC* gene was obtained through PCR amplification using the primer pair RNAi-sense-PtACC-F/R. Subsequently, a 400-bp anti-sense fragment containing a loop was amplified with the primer pair RNAi-anti-PtACC F/R. After that, by utilizing the ClonExpress® II One Step Cloning Kit C113–02 (Vazyme, Nanjing, China), the sense and anti-sense fragments were sequentially inserted into the C01-Mnsod1-RNAi plasmid (kindly provided by Fujian Agriculture and Forestry University). Eventually, the RNAi plasmid RNAi-PtACC was successfully constructed.

The C01-Mnsod1-OE plasmid (kindly provided by Fujian Agriculture and Forestry University) was double-digested with *Spe*I and *PspOM*I. Then, the CDS of the *PtACC* gene was inserted into the digested plasmid using the In-Fusion method, resulting in the successful generation of the overexpression plasmid OE-PtACC.

### 2.4 Transformation of *P. tuoliensis*

*P. tuoliensis* was genetically transformed utilizing the *Agrobacterium Tumefaciens*-Mediated Transformation (ATMT) method, as previously described in reference (Hou et al., 2019). The RNAi plasmid, RNAi-PtACC and the overexpression plasmids, OE-PtACC were individually introduced into *Agrobacterium tumefaciens* strain GV3101 (Coolaber, Beijing, China). Subsequently, these engineered *Agrobacterium* strains were used to transform the *P. tuoliensis* strain CCMSSC00489. The transformed strains were screened by being cultured on CYM agar plates supplemented with 30 μg/mL hygromycin and 400 μg/mL cefotaxime. To further validate the transformants, PCR was carried out using the primer pair Hyg-F/R, as detailed in Table 1, to amplify the hygromycin phosphotransferase gene (*Hpt*).

### 2.5 Real-time quantitative PCR (RT-qPCR)

The expression levels of *PtACC* in the strains were determined using real-time quantitative (RT-qPCR). Employing cDNA as the template and glyceraldehyde-3-phosphate dehydrogenase (*GAPDH*) as the internal reference gene, RT-qPCR was carried out on the ABI 7500 Fast Real-time PCR system (Applied Biosystems, Inc.) with the Taq Pro Universal SYBR qPCR Master Mix Q712–02 (Vazyme, Nanjing, China). The genes *GAPDH* and *PtACC* were amplified using primer pairs qGAPDH-F/R and qPtACC-F/R, respectively. The relative expression levels of the genes were calculated using the 2^-ΔΔCt^ method. Each sample was analyzed in triplicate.

### 2.6 Measurement of mycelial growth rate

The mycelial growth rates were determined individually on PDA, potato dextrose broth (PDB), and cottonseed husk medium. PDA plates were inoculated and then incubated at 25 °C in the dark for 7 days. After that, the diameter of the colony was measured, and the linear mycelial growth rate on PDA was calculated. The PDB flasks were inoculated and incubated at 25 °C in the dark with shaking at a speed of 150 rpm for 7 days. After that, the mycelia were harvested, dried at 60 °C, and then weighed. Finally, the mycelial biomass growth rate in PDB was calculated. The tubes contained with the cottonseed husk medium, which was composed of 78 % cottonseed husk, 20 % wheat bran, 1 % lime, and 1 % gypsum, with a moisture content of 65 %, were inoculated, and then incubated at 25 °C in the dark for 30 days. After that, the height of the medium colonized by the mycelium was measured, and the linear mycelial growth rate on the cottonseed husk medium was calculated.

### 2.7 Determination of Acetyl-CoA carboxylase activity

The activity ACC was determined using ACC Activity Assay Kit 1,012,405,222 (Boxbio, Beijing, China), following the manufacturer's instructions. The protein concentration within the samples was measured using the BCA Protein Concentration Assay Kit PC0020 (Solarbio, Beijing, China), adhering strictly to the provided protocol. The absorbance values were measured with a multimode plate reader infinite M200 Pro (Tecan, Mannedorf, Switzerland). One unit of specific ACC activity is defined as the amount of enzyme that consumes 1 nmol of NADH per minute per milligram of tissue protein.

### 2.8 Determination of total lipid content

The total lipids were extracted by the acid-heating extraction (Kong et al., 2010). First, 1 g of fresh mycelia were ground into powder using liquid nitrogen and then transferred into a 50-mL centrifuge tube. Subsequently, 4 mL of water was added and the mixture was thoroughly mixed. After that, 5 mL of an 8.3 mol/L HCl was added. The mixture was incubated in a water bath at 80 °C for 50 min, and it was mixed every 10 min. Once the sample was cooled to room temperature, 5 mL of 95 % ethanol was added and mixed well. Then, 10 mL of anhydrous ether was added, and the mixture was shaken for 1 min. After letting it stand for 20 min, the supernatant was carefully aspirated. Another 10 mL of anhydrous ether was added to repeat the extraction process. Finally, the total supernatant was transferred into a conical flask. The ether was completely evaporated by heating the flask in a water bath at 60 °C, and then the total lipids were weighed.

### 2.9 Analysis of fatty acid

The extracted lipids were converted into fatty acid methyl esters (FAMEs) using base-catalyzed transesterification (Ichihara & Fukubayashi, 2010). The lipid extract is transferred to a glass screw cap vial. 4 mL 2 % NaOH-methanol solution is added to the sample. The glass vial is incubated at 45 °C for 20 min in water bath. Then 4 mL 14 % BF_3_-methanol solution is added to the sample. The glass vial is incubated at 45 °C for another 20 min, and then is removed from the water bath and allowed to cool to room temperature. 3 mL of n-hexane is added to the sample, and shaken vigorously. Phase separation is achieved by standing undisturbed for 20 min. The upper organic phase containing the FAMEs is transferred to a disposable glass tube.

FAMEs were determined using the GC–MS systems Trace1310-ISQ (Thermo Fisher Scientific, USA) equipped with a TG-FAME (50 m × 0.25 mm × 0.20 μm) (Borrull et al., 2015). The injection temperature was 260 °C and oven temperature was programmed from 80 °C to 160 °C at a rate of 20 °C min^−1^, then to 230 °C at a rate of 5 °C min-1, and hold for 6 min. Helium was used at the carrier gas at a constant flow of 0.63 mL/min at a 5:1 split ratio. Temperatures of transfer line and ion source were held individually at 240 °C and 280 °C. The MS acquired data in the electronic ionization (EI) scan mode at 70 eV for the range of 60–900 amu after a solvent delay of 4 min.

## 3 Results

### 3.1 Transformation of *P. tuoliensis*

**Fig. 1:**
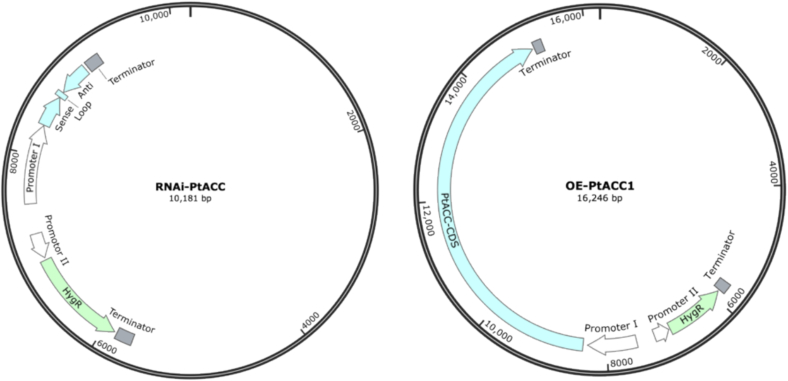
The schematic diagram of the plasmids.

Employing the ACC1 (Q00955) and HFA1 (P32874) of *S. cerevisiae* as queries separately, and using the genome (GCA_002147855.1) of *P. tuoliensis* as subjects respectively, tblastn analysis was conducted (Al-Feel et al., 1992; Bowman et al., 1997; Gao et al., 2018; Tian et al., 2024). It was found that there was only one *ACC* gene, namely *PtACC*, in the genome of *P. tuoliensis*. The 7366-bp DNA fragments and 6696-bp CDS fragments of the *PtACC* gene were successfully amplified via PCR. Based on plasmid C01-Mnsod1-OE, the RNA interference plasmid RNAi-PtACC and the overexpression plasmid OE-PtACC of gene *PtACC* were constructed respectively (Fig. 1).

**Fig. 2:**
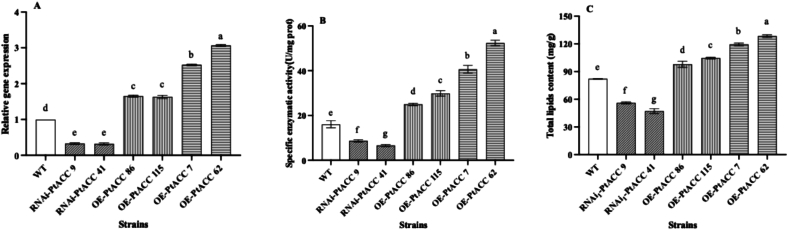
The relative gene expression of *PtACC* and specific activity of the ACC enzyme and the total lipid content within the strains. (A) The relative gene expression of *PtACC* of strains. (B) The specific activity of the ACC enzyme of strains. (C) The total lipid content within the strains. Values are the mean of three biological replicates ± SD (error bars). Different letters indicate a statistically significant difference (Dunnett's test, P<0.05).

The plasmid RNAi-PtACC was introduced into the *P. tuoliensis* strain CCMSSC00489 using ATMT method. A total of 31 transformed strains were successfully obtained. RT-qPCR analysis indicated that the relative expression levels of the *PtACC* gene in these 31 transformants were all lower than 65 % of those in the wild-type strain. The plasmid OE-PtACC was transformed into the *P. tuoliensis*. Thirty-three transformed strains were successfully obtained. RT-qPCR analysis demonstrated that the relative expression levels of the *PtACC* gene in these 33 transformants were 60 % to 210 % higher than those in the wild-type strain (Fig. 2，A).

### 3.2 Effect of *PtACC* expression level on ACC activity and total lipids content

The specific activity of the ACC enzyme and the total lipid content within the strains were systematically measured (Fig. 2, BC). When contrasted with the wild-type strains, the RNAi strains RNAi-PtACC 9 and RNAi-PtACC 41 showed a significant reduction in the relative expression levels of *PtACC*, decreasing by 67 % and 68 %, respectively. Correspondingly, the specific activities of the ACC enzyme in these RNAi strains declined by 46 % and 59 %, and the total lipid contents were 32 % and 43 % lower than those of the wild-type counterparts. Conversely, in comparison to the wild-type strain, the overexpression strains OE-PtACC 86, OE-PtACC 115, OE-PtACC 7, and OE-PtACC 62 manifested substantial increases in the relative expression levels of *PtACC*, with increase of 65 %, 63 %, 150 %, and 210 %, respectively. In tandem, the specific activities of the ACC enzyme in these overexpression strains increased by 56 %, 86 %, 154 %, and 227 %, and the total lipid contents increased by 19 %, 24 %, 45 %, and 56 %, respectively. Collectively, these results strongly indicate a profound and intricate correlation among *PtACC* expression level, the specific activity of the ACC enzyme, and the total lipid content in the strains.

### 3.3 Effect of *PtACC* expression levels on mycelial growth rate

**Fig. 3:**
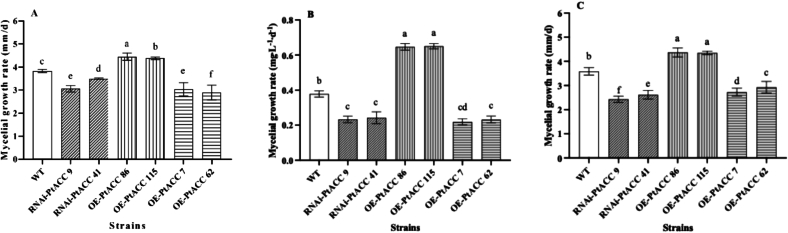
The mycelial growth rate of strains. (A) The mycelial growth on PDA medium; (B) The mycelial growth on PDB medium (C) The mycelial growth on cottonseed husk medium. Values are the mean of three biological replicates ± SD (error bars). Different letters indicate a statistically significant difference (Dunnett's test, P<0.05).

To explore the impact of the *PtACC* expression level on the mycelial growth rate, we measured the mycelial growth rates on PDA, PDB, and cottonseed hull media separately (Fig. 3). When compared with the wild-type strain, the RNAi strains RNAi-PtACC 9 and RNAi-PtACC 41 showed remarkable decreases in mycelial growth rates. Specifically, on the PDA medium, the growth rates of these two strains declined by 21 % and 8 % respectively. On the PDB medium, the mycelial growth rates dropped by 38 % and 35 %. Moreover, on the cottonseed hull medium, the reductions were 32 % and 29 % respectively.

In contrast, the overexpression strains OE-PtACC 86 and OE-PtACC 115 exhibited significant improvements in growth rates relative to the wild-type strain. On the PDA medium, the growth rates of these strains increased by 16 % and 14 % respectively. In the PDB medium, the mycelial growth rates increased by 70 % and 72 %. Similarly, on the cottonseed hull medium, the mycelial growth rates increased by 22 % and 21 % respectively. Interestingly, the mycelial growth rates of the overexpression strains OE-PtACC 7 and OE-PtACC 62 were unexpectedly lower than those of the wild-type strain. When cultivated on the PDA medium, the mycelial growth rates of OE-PtACC 7 and OE-PtACC 62 decreased by 21 % and 24 % respectively compared to the wild-type strain. In the PDB medium, their mycelial growth rates declined by 42 % and 38 %. On the cottonseed hull medium, the mycelial growth rates of these two overexpression strains also dropped, by 24 % and 18 % respectively.

Further analysis indicated that once the relative expression level of *PtACC* increased by more than 100 %, the mycelial growth rate consistently remained below that of the wild-type strain. These findings suggest that a decrease in the relative expression level of *PtACC* results in a slower mycelial growth rate. Generally, an increase in the relative expression level of *PtACC* promotes mycelial growth. Nevertheless, when this increase surpasses 100 %, it counterintuitively suppresses mycelial growth, which underscores the existence of a complex regulatory mechanism governing mycelial development.

### 3.4 Effect of *PtACC* expression level on fatty acid content

The total fatty acids of in the wild-type strain CCMSSC00489 and *PtACC* overexpression strain OE-PtACC 115 were analyzed utilizing GC–MS method. As shown in Table 2, a total of 21 kinds of fatty acids were detected in this study. The total fatty acid content of the wild-type strain CCMSSC00489 was 9.467 ± 0.281 mg/g, and the total fatty acid content of the overexpression strain OE-PtACC 115 was 24 % higher than that of the wild-type strain. Among them, linoleic acid (C18:2n6c) had the highest content, accounting for 73.19 % of the total fatty acids in the wild-type strain and 69.90 % of the total fatty acids in the OE-PtACC 115 strain. Except for three kinds of fatty acids, namely erucic acid (C22:1n9), tricosanoic acid (C23:0), and docosahexaenoic acid (C22:6n3), the contents of the remaining 18 fatty acids showed a significant disparity between the two strains. Specifically, the contents of these 18 fatty acids in the overexpression strain OE-PtACC 115 were notably higher than those in the wild-type strain CCMSSC00489. In particular, the content of docosanoic acid (C22:0) in the OE-PtACC 115 strain demonstrated a remarkable 213 % increase compared to that in the wild-type strain CCMSSC00489. These findings strongly suggest that the overexpression of *PtACC* can significantly elevate the contents of the majority of fatty acids.

**Table 2:** The fatty acid content of wild-type strain CCMSSC00489 and overexpression strain OE-PtACC 115.

Fatty Acid	CCMSSC00489(mg/g)	OE-PtACC 115(mg/g)
C14:0	0.019 ± 0.000 b	0.024 ± 0.000 a
C15:0	0.048 ± 0.002 b	0.086 ± 0.005 a
C16:0	1.248 ± 0.007 b	1.710 ± 0.005 a
C16:1	0.010 ± 0.000 b	0.019 ± 0.002 a
C17:0	0.038 ± 0.038 b	0.062 ± 0.004 a
C18:0	0.448 ± 0.007 b	0.562 ± 0.005 a
C18:1n9c	0.414 ± 0.010 b	0.495 ± 0.005 a
C18:2n6c	6.929 ± 0.205 b	8.229 ± 0.079 a
C20:0	0.014 ± 0.000 b	0.029 ± 0.002 a
C18:3n3	0.019 ± 0.000 a	0.019 ± 0.000 a
C20:1	0.014 ± 0.000 b	0.019 ± 0.000 a
C21:0	0.005 ± 0.000 b	0.010 ± 0.000 a
C20:2	0.010 ± 0.000 b	0.014 ± 0.000 a
C22:0	0.038 ± 0.002 b	0.119 ± 0.004 a
C20:3n3	0.029 ± 0.000 b	0.076 ± 0.007 a
C22:1n9	0.005 ± 0.000 a	0.005 ± 0.000 a
C20:4n6	0.014 ± 0.000 b	0.019 ± 0.000 a
C23:0	0.010 ± 0.000 a	0.010 ± 0.000 a
C24:0	0.143 ± 0.009 b	0.243 ± 0.005 a
C24:1	0.010 ± 0.000 b	0.019 ± 0.000 a
C22:6n3	0.005 ± 0.000 a	0.005 ± 0.000 a
Total	9.467 ± 0.281 b	11.771 ± 0.119 a

### 3.5 Effect of fatty acid on mycelial growth

**Fig. 4:**
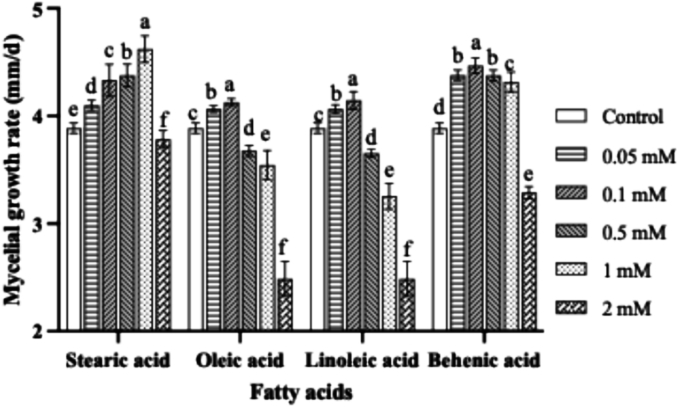
The effects of 4 fatty acids stearic acid, oleic acid, linoleic acid and behenic acid on the mycelial growth rate of wild-type strain CCMSSC00489. Values are the mean of three biological replicates ± SD (error bars). Different letters indicate a statistically significant difference (Dunnett's test, P<0.05).

In this study, four kinds of fatty acids with significant differences, namely stearic acid (C18:0), oleic acid (C18:1n9c), linoleic acid (C18:2n6c), and docosanoic acid (C22:0), were selected. Their impacts on the mycelial growth rate of the wild-type strain were investigated by adding them into the PDA medium. As illustrated in Fig. 4, all four fatty acids demonstrated the ability to enhance the mycelial growth rate of the wild-type strain at low concentrations. Specifically, when the concentration of stearic acid (C18:0) was 1 mM, the mycelial growth rate exhibited a 19 % increase relative to the control group; however, at 2 mM, it exerted an inhibitory effect on mycelial growth. For oleic acid (C18:1n9c), a concentration of 0.1 mM led to a 6 % growth enhancement compared to the control, while concentrations exceeding 0.5 mM inhibited growth. Linoleic acid (C18:2n6c) showed a 7 % increase in the mycelial growth rate at 0.1 mM relative to the control, but concentrations above 0.5 mM were inhibitory. Similarly, docosanoic acid (C22:0) promoted a 15 % growth increase at 0.1 mM compared to the control, yet concentrations higher than 2 mM suppressed mycelial growth. It is speculated that the overexpression of *PtACC* promotes the mycelial growth rate by increasing the content of fatty acids in the mycelium.

### 3.6 Effects of ammonium acetate, NaHCO_3_ and pH on mycelial growth

**Fig. 5:**
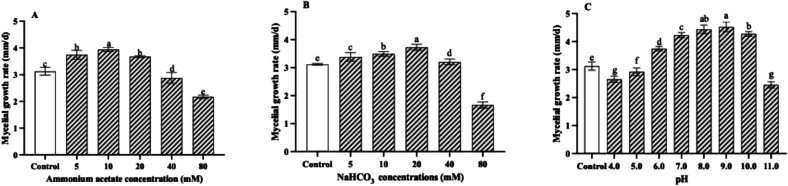
The effects of sodium acetate (A), NaHCO₃ (B), and pH (C) on the mycelial growth rate of wild-type *P. tuoliensis* strains on PDA medium. Values are the mean of three biological replicates ± SD (error bars). Different letters indicate a statistically significant difference (Dunnett's test, P<0.05).

The effect of ammonium acetate, NaHCO_3_ and pH on mycelial growth of the wild-type *P. tuoliensis* strain was evaluated on PDA medium. Results demonstrated that low concentrations of ammonium acetate (5 mM, 10 mM, and 20 mM) significantly promoted mycelial growth. The optimal concentration was observed at 10 mM ammonium acetate, which increased the growth rate by 26 % compared to the control. Once the concentration of ammonium acetate exceeds 40 mM, it will inhibit the growth of the mycelium (Fig. 5A). The low concentrations of NaHCO₃ significantly promoted the growth of the wild-type *P. tuoliensis* strain. The mycelial growth rate peaked at a NaHCO₃ concentration of 20 mM, showing a 19 % increase compared to the control without NaHCO₃ supplementation (Fig. 5B). *P. tuoliensis* wild type strain could grow on PDA medium within a pH range of 4–11, with the optimal at pH 9. At pH 9, the mycelial growth rate increased by 45 % compared to that in the control medium which is pH 5.5 without adjusting (Fig. 5C).

### 3.7 Effects of ammonium acetate, NaHCO_3_ and pH on lipid biosynthesis

**Fig. 6:**
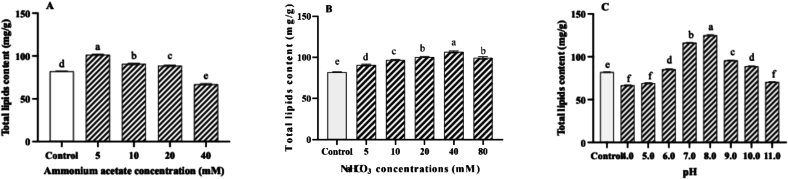
The effects of sodium acetate (A), NaHCO₃ (B), and pH (C) on the total lipid biosynthesis of wild-type *P. tuoliensis* strains on PDA medium. Values are the mean of three biological replicates ± SD (error bars). Different letters indicate a statistically significant difference (Dunnett's test, P<0.05).

Supplementation of ammonium acetate in the PDA medium can significantly alter the total lipid content in mycelia of the wild-type *P. tuoliensis* strain. The low concentrations of ammonium acetate (5 mM, 10 mM, and 20 mM) can significantly promote total lipid biosynthesis. The highest total lipid content was detected in the treatment supplemented with 5 mM ammonium acetate, demonstrating a significant 24 % increase compared to the control, which did not contain any ammonium acetate supplementation (Fig. 6A).

Supplementation of NaHCO₃ in the PDA medium which was adjusted into pH 7.0 with HCl, can significantly impact the total lipid content in mycelia. The NaHCO₃ ranged from 5 mM to 80 mM can increase the total lipid content. The maximum lipid accumulation occurred at 40 mM NaHCO₃, showing a 30 % increase compared to the control (Fig. 6B).

The pH of PDA medium significantly influenced the total lipid content in *P. tuoliensis* mycelia. Within the pH range of 6.0–10.0, the total lipid content was significantly higher than the control which is pH 5.5 without adjusting by HCl and NaOH. The maximum lipid accumulation occurred at pH 8.0, showing a 52 % increase compared to the control (Fig. 6C).

## 4 Discussion

### 4.1 ACC is a key enzyme in fatty acid biosynthesis

ACC catalyzes the conversion of acetyl-CoA into malonyl-CoA and is the first key enzyme in the de novo synthesis process of free fatty acids. Fatty acyl-CoA synthetase (FAS) then catalyzes condensation of acetyl-CoA and malonyl-CoA into saturated acyl-CoA. In the endoplasmic reticulum (ER), most of the acyl-CoA undergoes a desaturation reaction under the action of desaturase. Subsequently, saturated and unsaturated acyl-CoA are further assembled to form a variety of lipids, including glycerophospholipids, cholesterol esters, sphingomyelin, glycolipids, etc. (Greenwood et al., 2023; Konzock et al., 2022). These lipid substances play a crucial role in constructing the cell membrane structure and maintaining energy homeostasis.

In *S. cerevisiae* and *Y. lipolytica*, ACC, as the most crucial enzyme, directly determines the rate of fatty acid biosynthesis and the total lipids content (Duman-Özdamar et al., 2025; Mishra et al., 2023; Wang et al., 2020). The health promoting effects of lipids in edible mushrooms have already received substantial attention. Nevertheless, reports concerning the lipid biosynthesis are extremely scarce (Martínez-Ramírez et al., 2023; Sande et al., 2019). In this study, RNA interference and gene overexpression techniques were used to regulate the expression level of *PtACC* in *P. tuoliensis*. It was found that the change in the expression level of *PtACC* can directly affect the total lipid content in the mycelium. It suggests that ACC is also a key enzyme determining the fatty acid synthesis in *P. tuoliensis*.

### 4.2 Fatty acid synthesis is indispensable for the growth of *P. tuoliensis*

RNA interference-mediated reduction of *PtACC* expression significantly decreased the total lipid content and the mycelia growth rate of *P. tuoliensis*. Conversely, the overexpression of *PtACC* increased both parameters. These results indicate that lipid synthesis is essential for the growth of *P. tuoliensis*. Further studies showed that excessive overexpression of *PtACC* led to a significant increase in intracellular total lipid content, yet decreased mycelial growth rate. This could be due to the conversion of surplus fatty acids into triglycerides for energy storage after lipid synthesis meets basic fungal growth needs, such as building cell membrane structures, acting as precursor compounds for synthesis, and participating in signal transduction, etc. (Ferreira et al., 2018; Greenwood et al., 2023). Lipid synthesis is a highly energy-consuming metabolic process, which requires substantial amounts of acetyl-CoA, ATP, and NADPH, etc. (Lei et al., 2024; Zhang et al., 2021). Once the basic mycelial growth needs of *P. tuoliensis* are met, excessive lipid synthesis drains excessive energy, suppressing mycelial growth. A similar phenomenon has also been observed in *S. cerevisiae* when improving the synthesis of lipids by introducing *ACC1,* the growth rate of the cells will also decreased (Shi et al., 2014). In summary, the wild-type *P. tuoliensis* strains lack sufficient lipid synthesis capacity for optimal mycelial growth.

### 4.3 Supplying substrates of ACC in medium can promote lipid synthesis

In fungi, ACC is located in the cytoplasm and catalyzes the reaction: $\mathrm{Actyl}-\mathrm{CoA}+\mathrm{HC}O_{3}^{-}+\mathrm{ATP}\overset{\mathrm{ACCase}}{\to }\mathrm{Malonyl}-\mathrm{CoA}+\mathrm{ADP}+\mathrm{Phosphate}$. Both Actyl-CoA and bicarbonate ion are the substrates of ACC. Overexpressing genes such as *Acl* and *Cat2* to increase Acetyl-CoA in the cytoplasm, or overexpressing *Ca* to increase bicarbonate ion can both promote the synthesis of lipid (Chadwick & Lin, 2024; Liu et al., 2017; Wang et al., 2024; Xu et al., 2016; You et al., 2021).

In this study, supplementing the PDA medium with ammonium acetate which is converted to Acetyl-CoA by ACS in the cytoplasm can increases lipid synthesis and mycelial growth rate of *P. tuoliensis*. In addition, when the pH of the medium was kept constant, the supplementation of NaHCO₃ could also enhance the lipid synthesis and mycelial growth rate of *P. tuoliensis*. In addition, it suggested that the lipid accumulation may be involved in the mycelial response to environmental signals and primordium formation for *P. tuoliensis* (He et al., 2024). Therefore, lipid synthesis may be related not only to the growth of mycelium but also to the formation of fruiting bodies.

Carbon dioxide can affect the growth and development of edible mushrooms in multiple aspects. During the vegetative growth stage, increasing the carbon dioxide concentration within a certain range can promote mycelial growth for some edible mushrooms (Guo & Zhong, 2001; Zadražil, 1975). During the fruiting stage, CO_2_ promotes stipe elongation while inhibiting pileus expansion, which has been widely applied in the industrial production of *P. eryngii* and *Flammulina filiforms* (Lin & Lin, 2009; Liu et al., 2025). It suggested that the unsaturated fatty acids and their derivatives might take part in regulating the elongation growth of stipe of *F. filiforms* (Liu et al., 2019; Yao et al., 2019). Therefore, it is speculated that CO_2_ may promote the elongation of the stipe by affecting fatty acid synthesis.

This study determined that the optimal pH for *P. tuoliensis* mycelial growth is 8.0–9.0, with peak lipid synthesis at pH 8.0. Since mycelial respiration produces abundant CO₂, and $\mathrm{HC}O_{3}^{-}$ rises with pH below 10.33 (carbonic acid's pKα₂), the optimal growth pH of *P. tuoliensis* mycelium may correlate with lipid synthesis.

### 4.4 The poor lipid biosynthesis ability of *P. tuoliensis* may be the result of evolution

**Fig. 7:**
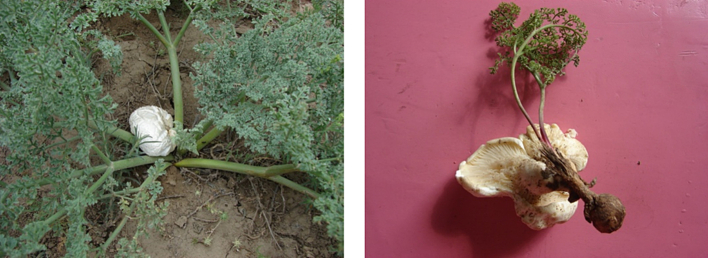
Photographs of *P tuoliensis*. Left: Wild *P. tuoliensis* in its natural habitat; Right: The harvested *P. tuoliensis* attaching to the base of *F. sinkiangensis*.

*P. tuoliensis* mainly grows at the base of living *Ferula sinkiangensis* or on the ground nearby under natural conditions. *P. tuoliensis* can grow together with *F. sinkiangensis* without causing its death (Fig. 7). it is speculated that it may form ectomycorrhiza. Artificial cultivation has shown that *P. tuoliensis* can complete its entire life cycle through saprophytism. It was shown that the ectomycorrhizal fungi originated evolutionarily from saprophytic fungi (Kohler et al. 2015; Martin et al., 2016; Genre et al., 2020). Some mycorrhizal fungi have lost partial degradation capacity and the ability to synthesize specific substances during evolution. Consequently, some carbon sources like carbohydrates and fatty acids are directly supplied by host plants (Jiang et al., 2017; Luginbuehl et al., 2017). The reduced lipid biosynthesis ability of wild-type *P. tuoliensis* is likely the result of long-term natural selection during evolution.

In nature, numerous ectomycorrhizal edible mushrooms, renowned for their delicious taste and health benefits, remain uncultivable, including *Tuber magnatum* and *Tricholoma matsutake* (Graziosi et al., 2024; Han & Su, 2025; Kim et al., 2024) *P. tuoliensis*, a mushroom with dual biotrophic and saprotrophic traits, shows enhanced mycelial growth upon increased fatty acid synthesis. This may offer potential strategies for domesticating other biotrophic edible mushrooms.

### 4.5 Adding fatty acids to the culture medium can increase the growth rate of mycelium

This study found that adding fatty acids to the medium can increase the mycelial growth rate of *P. tuoliensis*. Following uptake by *P. tuoliensis* mycelial cells, fatty acids either directly contribute to lipid biosynthesis or undergo β-oxidation to produce acetyl-CoA for de novo fatty acid synthesis. The specific metabolic route responsible for stimulating mycelial growth remains uncharacterized. It has been reported that supplementing fatty acids in the culture medium can effectively promote the mycelial growth of multiple edible fungi, such as *Ganoderma lucidum* and *Lentinula edodes* (Dai et al., 2014; Luo et al., 2008; Yao et al., 2010). These findings provide new insights into optimizing the formulation of edible fungus culture media. These results offer key insights for optimizing edible mushrooms culture media, driving cultivation innovation.

## 5 Conclusion

To address the slow mycelial growth of *P. tuoliensis*, this study manipulated *PtACC* expression via RNAi and overexpression methods. Reducing *PtACC* expression decreased total lipid content and mycelial growth, while increasing it had the opposite effect. Paradoxically, excessive *PtACC* expression inhibited growth. Supplementation of PtACC substrates, namely bicarbonate and ammonium acetate, also boosted lipid synthesis and mycelial growth. This indicates that *PtACC* is a key gene controlling the biosynthesis of lipids in *P. tuoliensis*. This provides new ideas for the domestication of edible mushrooms and the optimization of culture medium formulations, supplies theoretical foundations for molecular breeding, offers scientific frameworks for germplasm innovation, and delivers actionable solutions for expedited crop cultivation.

## CRediT authorship contribution statement

**Yu Tian:** Writing – original draft, Investigation, Data curation. **Suyue Zheng:** Writing – review & editing. **Jinwei Zhang:** Resources. **Qiang Chen:** Resources. **Ruiying Zhang:** Writing – review & editing, Funding acquisition.

## Consent to participate

Not applicable.

## Consent for publication

Not applicable.

## Ethics approval

Not applicable.

## Funding

This work was supported by the National key R&D Program of China (2023YFD1201600) and the Chinese Agriculture Research System (CARS-20).

## Declaration of competing interest

The authors declare that they have no known competing financial interests or personal relationships that could have appeared to influence the work reported in this paper.

## Data Availability

Data will be made available on request.
